# Lignosulfonic acid exhibits broad-spectrum anti-HIV and anti-HSV activity and has potential for microbicidal applications

**DOI:** 10.1186/1471-2334-14-S2-P27

**Published:** 2014-05-23

**Authors:** Geoffrey Férir, Sandra Claes, Stephanie Gordts, Graciela Andrei, Robert Snoeck, Dominique Schols

**Affiliations:** 1 Rega Institute for Medical Research, Leuven, Belgium

## 

Multiple clinical studies show the importance of genital HSV-2 infections in women on the sexual transmission of HIV-1 and vice versa. Therefore, it is important to develop molecules with potent dual antiviral activity to reduce sexual transmission of these life-treating pathogens. Here, we describe that the polyanionic compound lignosulfonic acid (LA; MW: 8000), which belongs to the family of lignin-derived macromolecules, is a cheap byproduct that is formed during the conversion of wood pulp into paper. LA exhibits low toxicity (CC50: >62.5 µM) and potent broad-spectrum anti-HIV activity (IC50s: 0.10 - 0.34 µM), irrespective of the viral coreceptor tropism. In addition, it inhibits the formation of giant cells between HIV-1 persistently infected cells and non-infected CD4 target T cells (IC50: 0.23 µM).

HSV-2 is not solely an epitheliotropic virus, as it also infects CD4 T cells, macrophages and MDDCs. We show that LA is able to inhibit HSV-2 infection in HIV-1 susceptible T cells in the lower µM-range (IC50: 0.78-0.82 µM). In addition, LA blocks viral replication of dual HIV-1 and HSV-2 infected cells with comparable potency (IC50: 0.71 µM).

DC-SIGN plays an important role in the sexual transmission of HIV and of HSV. We could show that LA (12.5 µM) has no effect on the capture of HIV by Raji.DC-SIGN cells, however it inhibited further transmission to uninfected CD4 T cells (IC50: 1.2 µM). Comparable observations were made with HSV-2 using MDDCs and MT-4 cells (IC50: 1.1 µM). We investigated also its potential side-effects and stimulatory effects. PBMCs were exposed to high amounts of LA and the expression of CD25, CD69 and HLA-DR on CD4+ T cells was measured. No significant increased effects were seen for each activation marker, while the mitogenic lectin PHA stimulated. Finally, LA did not harm the vaginal lactobacilli and synergistic activity was observed with acyclovir, PRO2000 and the lantibiotic LabyA1. In conclusion, lignosulfonic acid has potential for a microbicidal agent.

